# A Rare Case of Small Intestinal Cancer With Uterine Metastasis After Surgery for Ovarian Metastasis, Diagnosed Using Immunostaining

**DOI:** 10.1155/2024/8551816

**Published:** 2024-08-10

**Authors:** Sachiko Nagao, Motoki Matsuura, Shoko Kurokawa, Masato Tamate, Taishi Akimoto, Tsuyoshi Saito

**Affiliations:** Department of Obstetrics and Gynecology Sapporo Medical University, Sapporo, Hokkaido, Japan

**Keywords:** cancer of the ileum, diagnostic techniques, metastasis, surgery, uterus

## Abstract

Uterine metastases from extragenital sites are rare. We present a case of a woman who had undergone surgery for small intestinal cancer and subsequently developed metastases in her left ovary and uterus. A nulliparous woman in her 50s underwent laparoscopic partial small bowel resection with lymph node dissection for small intestinal cancer. Five months later, computed tomography (CT) revealed a left ovarian tumor and ascites. She underwent bilateral adnexectomy and adjuvant chemotherapy, and the ovarian tumor was diagnosed as a small intestinal cancer metastasis. Two years after the small intestinal cancer surgery, a positron emission tomography (PET)-CT scan revealed a uterine accumulation. Cervical cytology was negative for intraepithelial lesion or malignancy. Endometrial histology showed an adenocarcinoma of the uterus. The patient underwent total abdominal hysterectomy followed by adjuvant chemotherapy. Histopathology and immunohistochemistry of the uterine tumor revealed that it was a metastasis of small intestinal cancer (Cytokeratin 7 [CK7] [−], Cytokeratin 20 [CK20] [+], Special AT-Rich Sequence-Binding Protein 2 [SATB2] [+], Paired Box Gene 2 [PAX2] [−], and estrogen receptor [ER] [−]). In patients with cancer, histopathology and immunohistochemistry are important for distinguishing between primary and metastatic tumors and for guiding the choice of treatment.

## 1. Introduction

Compared with metastatic ovarian tumors, metastatic uterine tumors are rare. A study by Karpathiou et al. [1] found that metastatic tumors to gynecological organs involve the ovary in 50% of cases, whereas the endometrium and myometrium are involved in only 3.6% and 10.7% of cases, respectively. The primary sites reported for metastatic uterine tumors include the colon, stomach, breast, malignant melanoma, and lungs [2, 3]. Small bowel cancer is rare, accounting for less than 0.5% of all malignant tumors and 5% of all gastrointestinal malignant tumors.

This report describes a rare case of small bowel cancer metastasizing to the ovary and uterus in a patient who had undergone surgery for small bowel cancer.

## 2. Case Presentation

**Figure 1:**
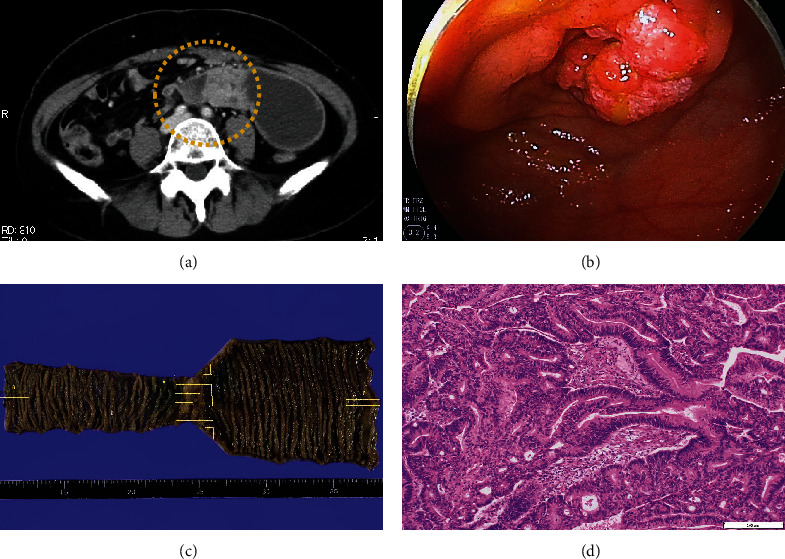
Small bowel cancer findings. (a) Contrast-enhanced computed tomography of the abdomen showing neoplastic lesions in the small bowel. (b) Capsule endoscopy showing a Type 2 tumor, 2 cm in diameter. (c) Surgical specimen showing a Type 2 tumor measuring 25 × 10 mm. (d) Histopathology showing columnar cell growth forming fused, cribriform, and papillary ducts, characteristic of moderately differentiated adenocarcinoma (hematoxylin and eosin stain; space bar: 100 *μ*m).

**Figure 2:**
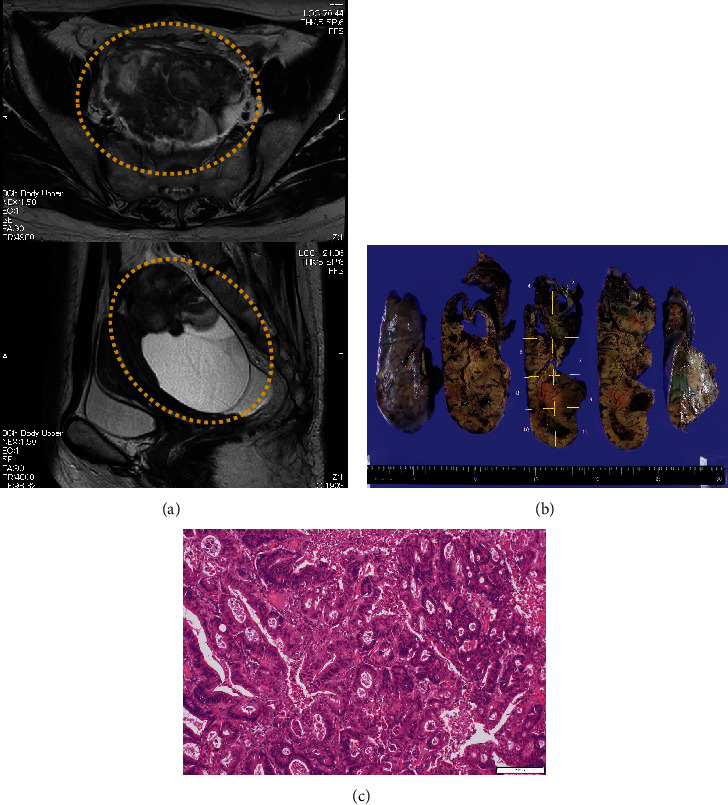
Findings of the tumor in the ovary 5 months after small bowel cancer surgery. (a) T2-weighted magnetic resonance imaging (MRI) showing left ovarian enlargement. (b) Surgical specimen of the left ovary. The left ovary showed a grayish-white to yellowish-white, substantial lesion with cyst formation. (c) Histopathology of the left ovary showing findings similar to the small bowel cancer (hematoxylin and eosin stain; space bar: 100 *μ*m).

**Figure 3:**
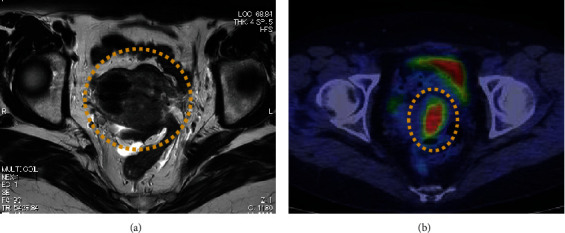
Imaging showing a tumor in the uterus 24 months after small bowel cancer surgery. (a) Contrast-enhanced computed tomography (CT) showing uterine enlargement. (b) Positron emission tomography (PET)/CT showing accumulation in the uterus.

**Figure 4:**
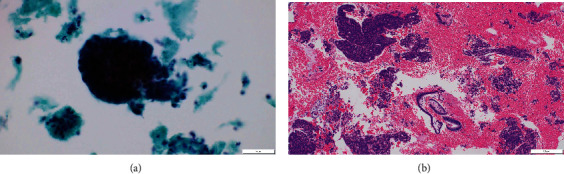
Endometrial findings of the tumor in the uterus. (a) Endometrial cytology showing clusters of atypical cells with enlarged nuclei, irregular karyotype, and distinct nuclei on a hematogenous background; prominence of irregular stacking and disarrangement of nuclei, consistent with adenocarcinoma (space bar: 50 *μ*m). (b) Endometrial histology showing atypical cells proliferating solidly within the stromal tissue, suggesting poorly differentiated carcinoma (hematoxylin and eosin stain; space bar: 200 *μ*m).

**Figure 5:**
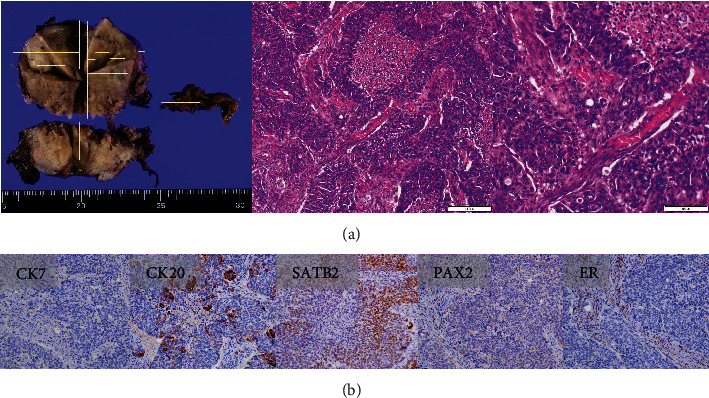
Characteristics of the uterine tumor. (a) Left, uterus; middle and right, histopathology showing moderate to poorly differentiated adenocarcinoma spreading extensively throughout the uterus with lymphatic invasion (hematoxylin and eosin stain; space bar: 200 *μ*m). (b) (left to right) immunostaining for CK7 (−), CK20 (+), SATB2 (+), PAX2 (−), and ER (−). These results are typical of gastrointestinal cancer rather than uterine cancer (Table 1) and led to the diagnosis of uterine metastasis of small bowel cancer rather than primary uterine cancer. CK7, Cytokeratin 7; CK20, Cytokeratin 20; ER, estrogen receptor; PAX2, Paired Box Gene 2; SATB2, Special AT-Rich Sequence-Binding Protein 2.

The patient was a nulliparous woman in her 50s who had undergone menopause at the age of 49 years old. Her father had colon cancer. She underwent laparoscopic partial small bowel resection and regional lymph node dissection for small bowel cancer in our gastroenterological surgery department. Histopathological examination revealed a Type 2 tumor measuring 25 × 10 mm with high columnar cell growth forming fused, cribriform, and papillary ducts on histopathology. The tumor was diagnosed as a moderately differentiated adenocarcinoma, pT4, pN2, cM0, and Stage IIIB (Figure 1). Five months after the surgery, a follow-up contrast-enhanced computed tomography (CT) scan revealed a left ovarian tumor and ascites, and the patient was referred to our department. A magnetic resonance imaging (MRI) scan revealed a left ovarian tumor with heterogenous enhancement, measuring 107 × 57 mm (Figure 2). No obvious lesions were noted in the uterus. The enhanced portion showed a diffusion abnormality in the left ovary suggestive of malignancy. A bilateral abdominal adnexectomy was performed, and histopathological examination revealed the same histopathology as the previous small bowel cancer (Figure 2). Immunostaining was negative for Cytokeratin 7 (CK7), positive for Cytokeratin 20 (CK20) and Special AT-Rich Sequence-Binding Protein 2 (SATB2), and negative for estrogen receptor (ER) and Paired Box Gene 2 (PAX2), leading to a diagnosis of metastatic recurrence of small bowel cancer. The patient received 12 cycles of mFOLFOX as postoperative chemotherapy. A positron emission tomography (PET)/CT scan 24 months after the small bowel cancer surgery revealed accumulation in the uterus and obturator lymph nodes (Figure 3), and the patient was referred back to our department with suspected uterine metastasis of the small bowel cancer. Her symptoms included a small amount of genital bleeding. Tests of tumor markers showed elevated levels of CA19-9, carcinoembryonic antigen (CEA), and CA125. Cervical cytology was negative for intraepithelial lesion or malignancy, but endometrial cytology revealed clusters of atypical cells with enlarged nuclei, an irregular karyotype, and distinct nuclei on a hematogenous background, with prominent irregular stacking and disarrangement of nuclei, leading to a diagnosis of adenocarcinoma. Endometrial histology revealed atypical cells proliferating solidly within the stromal tissue, a finding that suggested poorly differentiated carcinoma (Figure 4). Immunostaining of the atypical cells was negative for CK7, partially positive for CK20, and negative for ER, indicating metastatic small bowel cancer rather than a primary malignant uterine tumor. Although pelvic and para-aortic lymph node dissection is generally performed in primary malignant uterine tumors, considering the long duration of the surgery and the high risk of perioperative complications such as lymphedema, a total abdominal hysterectomy and pelvic lymph node biopsy were performed without lymph node dissection. Macroscopically, the tumor had grown to replace the myometrium and was also exposed on the serosal surface. Histopathological examination revealed moderate to poorly differentiated adenocarcinoma with extensive spread throughout the uterus and lymphatic invasion. The ascites cytology was negative. Immunostaining was negative for CK7, positive for CK20 and SATB2, and negative for ER and PAX2; therefore, the patient was diagnosed with a uterine metastasis of small bowel cancer (Figure 5 and Table 1). The patient is currently undergoing FOLFIRI+BEV therapy.

## 3. Discussion

Small bowel cancer is rare, accounting for less than 0.5% of all malignant tumors and 5% of all gastrointestinal malignant tumors. There is no standard treatment, but primary tumor resection is commonly performed for Stages I–III, and systemic chemotherapy is commonly administered for Stage IV or postoperative recurrence. In Japan, FOLFOX therapy for Stage IV or postoperative recurrence has been covered by insurance since September 2018.

Uterine metastases due to extragenital primary tumors are rare [1]. Primary sites reported include the colon, stomach, breast, malignant melanoma, and lungs [2, 3]. In Japan, several cases of metastasis of gastric cancer to the uterus have been reported [4–6]. In recent years, the number of cases of uterine metastasis is thought to have been increasing as the incidence of colon and breast cancer increased. The most common symptom of uterine metastasis is irregular bleeding, but it is sometimes asymptomatic [7], and this case was discovered during follow-up imaging examination of the patient after treatment for small bowel cancer. The reasons for the rarity of uterine metastasis include that lymph flows away from the uterus and because fibromuscular tissue is unfavorable for metastatic tumors [8].

Cytology and histology tend to be negative because metastatic tumors often invade the uterine stroma and myometrium and are rarely exposed to the mucosal surface. Cases with invasion of the cervix should be distinguished from cervical adenocarcinoma.

In the case of this patient, pathology specimens from the primary lesion in the small bowel showed a moderately differentiated adenocarcinoma, but the metastatic lesion in the uterus showed a moderate to poorly differentiated adenocarcinoma. Histologic changes in metastatic lesions, especially dedifferentiation to poorly differentiated carcinoma, are common after chemotherapy. This is hypothesized to be because only resistant cancer cells remain after chemotherapy [9].

## 4. Conclusion

In this patient, because the tumor was exposed on the endometrium, endometrial cytology revealed malignant cells, and immunostaining on endometrial histology enabled the determination of the primary site. The high possibility of a metastatic tumor was taken into consideration in planning the surgery, and the patient was able to smoothly transition to treatment for recurrent small bowel cancer. A careful examination taking the possibility of metastatic tumors originating from other organs into account is useful for making policy decisions and treatment choices regarding patient management. In addition, uterine metastasis from small bowel cancer is an extremely rare condition, so in the initial surgery, bilateral adnexectomy was performed because no signs of uterine involvement were present at the time and the risk of uterine metastasis was considered to be low; however, a uterine metastasis developed subsequently. Although bilateral adnexectomy alone is often performed when ovarian metastasis is diagnosed, some surgeons perform a prophylactic hysterectomy based on the possibility that a uterine metastasis may be found later, as in this case.

## Figures and Tables

**Table 1:** Immunostaining results in uterine and gastrointestinal cancer.

**Tumor marker**	**Uterine tumor in the patient**	**Uterine cancer**	**Gastrointestinal cancer**
CK7	−	+	+ or −
CK20	+	−	+
SATB2	+	−	+
PAX2	−	+	−
ER	−	+	−

*Note:* The immunostaining results of the uterine tumor did not correspond with the markers found in uterine cancer but were typical of those found in gastrointestinal cancer. This led to the diagnosis of uterine metastasis of small bowel cancer rather than primary uterine cancer.

Abbreviations: CK7, Cytokeratin 7; CK20, Cytokeratin 20; ER, estrogen receptor; PAX2, Paired Box Gene 2; SATB2, Special AT-Rich Sequence-Binding Protein 2.

## Data Availability

The data that support the findings of this study are available on request from the corresponding author. The data are not publicly available due to privacy or ethical restrictions.
